# Creating a multi-linked dynamic dataset: a case study of plant genera named for women

**DOI:** 10.3897/BDJ.11.e114408

**Published:** 2023-12-06

**Authors:** Sabine von Mering, Lauren Maria Gardiner, Sandra Knapp, Heather Lindon, Siobhan Leachman, Carmen Ulloa Ulloa, Sarah Vincent, Maria S. Vorontsova

**Affiliations:** 1 Museum für Naturkunde, Leibniz Institute for Evolution and Biodiversity Science, Berlin, Germany Museum für Naturkunde, Leibniz Institute for Evolution and Biodiversity Science Berlin Germany; 2 Cambridge University Herbarium, Cambridge, United Kingdom Cambridge University Herbarium Cambridge United Kingdom; 3 Natural History Museum, London, United Kingdom Natural History Museum London United Kingdom; 4 Royal Botanic Gardens Kew, Richmond, United Kingdom Royal Botanic Gardens Kew Richmond United Kingdom; 5 Independent researcher, Wellington, New Zealand Independent researcher Wellington New Zealand; 6 Missouri Botanical Garden, St. Louis, United States of America Missouri Botanical Garden St. Louis United States of America; 7 Royal Botanic Gardens, Kew, London, United Kingdom Royal Botanic Gardens, Kew London United Kingdom

**Keywords:** women botanists, botanical names, eponymy, eponyms, etymology, naming, nomenclature, gender studies, Wikidata, International Plant Name Index (IPNI), Tropicos, Biodiversity Heritage Library (BHL), Linked Open Data (LOD), Open Science

## Abstract

**Background:**

A discussion on social media led to the formation of a multidisciplinary group working on this project to highlight women’s contributions to science. The role of marginalised groups in science has been a topic of much discussion, but data on these contributions are largely lacking. Our motivation for the development of this dataset was not only to highlight names of plant genera that honour women, but to enrich this information with data that would allow the names, roles and lives of these women to be shared more widely with others, both researchers and data sources like Wikidata. Amplification of the contributions of women to botany through multiple means will enable the community to better recognise and celebrate the role of this particular marginalised group in the history and development of science.

**New information:**

The innovative approach of our study resulted in a dataset that is dynamic, expansive and widely shared. We have published a static dataset with this paper and have also created a dynamic dataset by linking flowering plant genera and the women in whose honour those genera were named in Wikidata. This concurrent addition of the data to Wikidata, a linked open data repository, enabled it to be enriched, queried and proactively shared during the whole process of dataset creation and into the future. This innovative workflow allowed wide, open participation throughout the research process. The methodology and workflows applied can be used to create future datasets celebrating and amplifying the contributions of marginalised groups in science.

## Introduction

Gender stereotypes in science often result in the exclusion of women from scientific professions (UNESCO 2007) and even when women are active scientifically, their work tends to be credited less than that of their male colleagues (Ross et al. 2022). The advent of literature databases and citation indices means that disparities in gender can be revealed for scientific practice currently and in the recent past, but delving into the history of science can be more challenging (Lindon et al. 2015, Lindon et al. 2023). One way in which the visibility of women can be demonstrated is through the taxonomic practice of eponymy - the naming of taxa for people. People are honoured in many ways through eponymy; streets, monuments, parks and cities have all been given names to celebrate individuals. Eponymy is an established tradition in botany. In his *Critica Botanica*, Linnaeus (1737) articulated rules for the naming of plants, some of them related to the sorts of people plants should, or should not, be named for. Linnaeus’ “aphorisms” clearly reflect the society of his time - rulers are kings and deities are gods. Women and other marginalised groups are largely invisible in the world of 18th-century plant naming.

Plant names at all taxonomic levels may be named for people - creating eponyms at genus, species and infraspecific levels. Here, we focus on the names of flowering plant genera - there are fewer genera than there are species (ca. 16,000, with approximately 40 new genera described annually, versus ca. 450,000 species, with approximately 2,000 added annually; Bebber et al. 2010) and tradition has it that a genus name carries more “weight” and status than that of a species name.

Recently, some authors (Guedes et al. 2023) have suggested that all eponyms should be changed and that they should not be permitted in the future, due to the preponderance of names of colonial scientists in the names of terrestrial African vertebrates. Views about this differ (e.g. Smith and Figueiredo 2021, Mosyakin 2022, Antonelli et al. 2023, Garbino 2023, Jost et al. 2023, Pethiyagoda 2023) and in the community working with algae, fungi and plants, these topics will be discussed at the upcoming International Botanical Congress in Madrid in 2024, where proposals to change the *International Code of Nomenclature for algae, fungi, and plants* (hereafter ICN, Turland et al. 2018) will be debated (Hammer and Thiele 2021, Smith and Figueiredo 2021).

Lindon et al. (2015) demonstrated that female authors of botanical names represent about 12% of the total number of authors and women published approximately 8% of all botanical species names. They clearly showed that the disparity in gender in authorship has decreased over time, suggesting that women are today as productive as authors as their male colleagues. Another way to look at women’s contribution to — or celebration as contributors to — botany is to explore eponyms themselves and what kinds of plants were and are named for women.

Our work in creating this dataset was motivated by the desire to amplify the contribution of women to botany through eponymy. We were inspired by the “Funk List” which has helped make women scientists and their contributions more visible (Kapsalis 2019, Wagner and Wen 2023) and by recent work in creating identifiers for people to uncover contributions that have hitherto remained hidden (Groom et al. 2020, Groom et al. 2022). Here, we deliberately have not compared the differences between eponyms based on female names and those based on the names of men - our intention is to amplify women’s contributions to botany through a unique dataset that has developed as Open Science by linking and adding to multiple other data sources as the work continued.

Social media played a large part in coalescing the efforts described in this paper, with tweets seeking information about flowering plants named for women being widely disseminated and responses widely shared. Initially, it was thought of as being a simple question that perhaps had a simple answer. Subsequent conversations, however, revealed that a dataset, like the one needed to answer this simple question, did not exist and that elements of such a dataset involved many disparate institutions and data sources. Bringing these data elements together involved a complex and innovative workflow and this paper documents that process.

Our principal objectives for this work were to: 1) create a dataset of Linked Open Data (LOD) that amplified the names of women celebrated through eponymy at the generic level, 2) verify and document eponymy for flowering plant genera thought to be named for women or female mythical beings and 3) document a methodology for developing an enriched Linked Open Dataset that benefits from both push and pull - pushing data to and pulling from a wide variety of sources to create new resources for future research.

## General description

### Purpose

In May 2021, a social media discussion and investigations for a book about plants named for people (Knapp 2022) sparked our interest and further investigations on taxa named for women. A tweet by one of the authors (CUU) about the plant genus *Meriania* Sw., honouring naturalist and artist Maria Sibylla Merian, received some feedback about other genera and led to the questions: ‘how many plant genera were actually named after women’ and ‘who are or were these women’? However, there was no simple way to answer these questions with the data available at the time. A standardised list of plant genera, their eponymy, the gender and occupations of the person honoured did not exist. Compiling such a dataset was difficult as the relevant information is stored in various places (databases, literature etc.) and in different formats.

The conversation on social media led to the formation of this international working group of co-authors with an interest in the topic and different areas of expertise: botanical nomenclature, botanical history, data management and integration, data analysis and visualisation. It was also fundamental that members of the group had institutional access to the specialised botanical literature required to verify the original descriptions of plant genera (protologues) when these were not available online. Our different backgrounds and multidisciplinarity, involving researchers active in different institutions and a Wikimedian, were crucial for the success of our informal working group and we started referring to ourselves as the “women genera” group.

We met regularly and mostly virtually over a period of more than two years and worked synchronously and asynchronously using digital tools. This project aimed to create an open and dynamic dataset connecting flowering plant (angiosperm) genera with the women and female beings (from mythology or mythical stories) in honour of whom the genera were named. The group was committed to Open Science and thought strategically about how to share the data created or improved through the research undertaken. We prioritised proactively sharing the data generated as widely as possible during, rather than after, the research process, adding and linking it to multiple databases and sources (push-pull), such as the International Plant Name Index (hereafter IPNI), Tropicos®, Wikidata, Bionomia and the Biodiversity Heritage Library (hereafter BHL). Preliminary project results were presented at several conferences informing relevant communities about the approach and progress of our work (Knapp et al. 2022, Gardiner et al. 2022, Leachman et al. 2023). Communication at scientific meetings and via social media created more visibility for the project and generated new questions and ideas to explore.

## Sampling methods

### Study extent

**Methods, tools and workflow**

**Linked resources**

Wikidata was the central hub for this project. Wikidata is an open, collaborative and multilingual knowledge base providing structured data that can be read and edited by humans and machines (Vrandečić and Krötzsch 2014, Shafee et al. 2023). As a hub for other identifiers, it plays a central role in semantic linking and enriching of data (e.g. Groom et al. (2020), Groom et al. (2022), Page (2022), Page (2023)). Wikidata properties mentioned in the text are given in *italics* followed by the Wikidata entity link.

One advantage of using Wikidata as the hub for the project was the ability to use the Wikidata Query service to answer specific questions needed for improvement of the dataset. Pulling information from multiple sources and verifying and referencing each one to build the new dataset is time-consuming, but each interaction allowed us to query the resulting data (or subsets of it) with the Wikidata Query Service using SPARQL. For an example of such a SPARQL query, see Suppl. material 1. Wikidata also provides a the so-called Query Builder, a tool that empowers users with minimal coding/scripting knowledge to build simple Wikidata queries (e.g. botanists from a particular geographic area or plants named for fictional characters).

**Figure 1.**
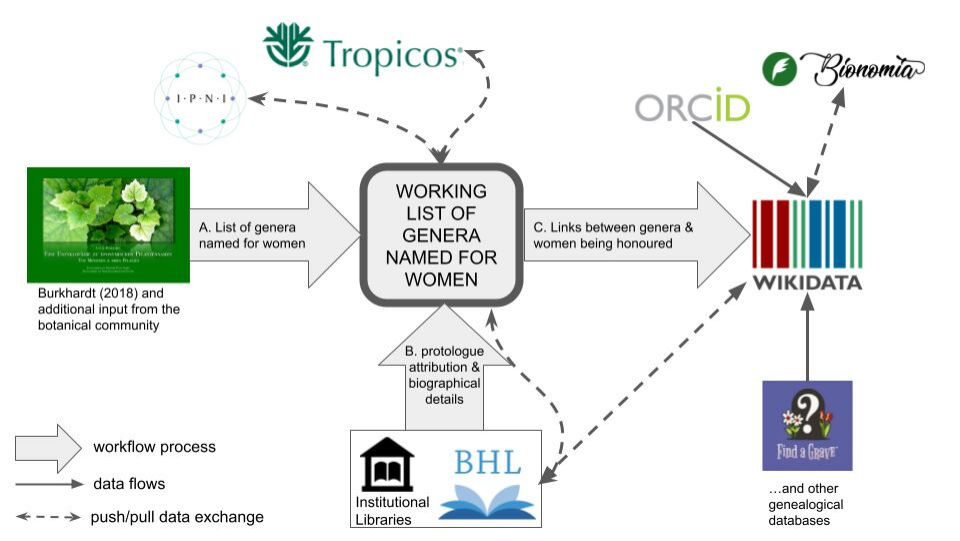
The principal linked resources used in the dataset creation process and the flow or push and pull of data between them. Arrowheads indicate flow of information, lines with two arrows indicate where information was both pulled from outside sources and pushed back to improve those sources during the course of the project. The main resources used for the bulk of the work are highlighted, others not pictured here are mentioned in the text or in the dataset itself.

The key for answering the original questions posed were links between various elements in the dataset. The principal linkages used in creation of the dataset are shown in Fig. 1.

Linking plant genera and the women for whom they were named was the central link in our workflow. Once Wikidata items existed for all plant genera and all women (or female beings) honoured, these items were linked using the property *named after*. Each Wikidata item for a plant genus honouring a female was linked via a referenced statement to the item of the honoured woman or female being. It was this central link that allowed us to address our initial question.

To find information on eponymy contained in the original places of publication for generic names (protologues), we searched botanical databases, such as IPNI and Tropicos that often provide links to BHL. In other cases, we used openly accessible providers of digital publications, such as Google books, Hathi project and other digital libraries and websites that provide free access to such publications. BHL was invaluable to this research project as the majority of protologues (457, i.e. over 60%) were openly accessible through this digital library. Some texts were either in copyright or were missing from BHL as well as other providers of digital publications. Where we were unable to freely access the protologues digitally, we consulted the libraries accessible to us at our own institutions. During this process, we collated a list of all those publications that appeared to be in the public domain, but were missing from BHL. We passed on this list to BHL and requested these “missing” texts be scanned and added to BHL for the benefit of everyone. As of writing, BHL staff are working on this request.

Women for whom plant genera were named include both female beings from mythology (mythical women) and those who are documented to have actually lived; we termed these non-mythical women (see section on People below). For non-mythical women involved in collecting or identifying specimens, we created or improved their profiles in Bionomia and attributed specimens collected or identified by them. For deceased women, we made their Bionomia profiles public and added their Bionomia identifier to the women’s Wikidata items. If the woman were living and had registered for an ORCID, we were able to use this identifier to attribute specimens to her in Bionomia. However, while profiles of deceased people can be made public, living people have full control over their Bionomia profile and decide for themselves to make it public or remain private.

Over the course of our work, we found small errors in publicly available datasets that are used by many botanists. These publicly available datasets were created over decades using a variety of methods (Croft et al. 1999). They are immensely useful and the community is invited to improve them. Corrections or newly-researched information on taxon authors, protologues and eponyms was sent together with the corresponding references to IPNI and Tropicos and updated in these databases accordingly. As a result of our research into the protologues, we frequently contacted the IPNI editors with corrections (e.g. dates or page numbers) or enhancements, such as standardising author names or adding links to protologues that were difficult to track down. We also consulted the IPNI team to discuss difficult nomenclatural issues. We edited the Tropicos database directly with corrections and improvements. Missing IPNI and Tropicos genus identifiers were added to the Wikidata items of the respective genera.

**Generic names of plants**

The starting point for our list of plant genera named for women (both mythical and non-mythical) was the “Index of Eponymic Plant Names - Extended Edition” by Lotte Burkhardt (Burkhardt 2016, Burkhardt 2018) available online as a searchable PDF file. We manually extracted all genera honouring women (Fig. 1, Workflow A) and created a simple table. Burkhardt published an updated version in 2022 and kindly provided us with a list of additional genera included in that edition (Burkhardt 2022). This dataset was supplemented with data from IPNI (2023) and other sources including Baines (1981), Quattrocchi (1999), Charters (2015) and Mari Mut (2021), as well as suggestions received from colleagues and generated from our own research. We extracted the available etymology data in IPNI for all included genera to obtain any relevant information on the origin of a name. In some cases, we contacted the authors directly via email to ask who their name was honouring.

Other genera named for women were found using the code of nomenclature governing plant naming (ICN; Turland et al. (2018)), where clear rules are set out, in, for example, Rec. 60B. This recommendation sets out good practice for forming generic names by adding -*a*, -*ea* or -*ia* to the person’s name and Note 2 describes how to modify names to name different genera after the same person using a prefix or suffix. Recommendation 60H suggests providing the etymology or source of the name in the protologue when publishing the name of a new plant at any rank to give some indication of the derivation of the name.

Our dataset covers angiosperm genera (flowering plants) named after perceived women and female beings. Hybrid and fossil genera were excluded as well as names that were not validly published, while illegitimate names were included (see ICN for an explanation of these nomenclatural terms; Turland et al. (2018)). For genera published by French naturalist Constantine Rafinesque, we excluded those that appeared to have been named after nymphs that did not have any clear reference to Greek or Roman mythology. It has been suggested that these names were probably “made out of thin air” (Bernhardt 2008, Mari Mut 2019).

For all genera, the protologues were reviewed to confirm the etymology or eponymy. Newer publications usually include a specific “etymology” section explaining the origin of the assigned, in most cases, honorific name (Agosti et al. 2022). Sometimes a specific dedication text is given stating the role or specific achievements of the person. In other cases, the name of the person was not specified in the dedication, but could be indirectly implied, for example, as a collector (see *Clemensia* Merr.) or in the name of the type species (e.g. *Dorotheatalbotii* Wernham). Dedications were copied in the original language into our working sheet accompanied at times with a translation. Where possible when citing the protologue in support of the *named after* statement in Wikidata, we quoted the protologue dedication text. For about a third of the genera (223), there was no ascription given in the protologue. When the etymology was not stated in the protologue, but by Burkhardt (Burkhardt 2016, Burkhardt 2018, Burkhardt 2022), we used these references to support the *named after* statement in Wikidata.

To be able to answer our research questions, we collected the following information as structured data: the year and place of publication (nomenclatural reference), the author(s) of the name and the digital link to the protologue or original publication (if available online) plus information on the honoured women (see section “People” below). A full list of data fields is given in the Data dictionary (see column field and description below).

Several genera were named after their similarity to other genera that were named for women. We classified such eponymy as “indirect”, but included them in our study since the derived eponyms are also honouring women. In Wikidata, these have been noted by adding *named after* statements linking the item of the new genus to both the genus after which it was named and to the woman/female being who was originally honoured and, therefore, honoured in the new genus indirectly. This information on indirect eponymy was entered in our data table in the column *eponymy-derived-indirectly*. For example, the genus *Afrofittonia* Lindau is named for the genus *Fittonia* Coem. and, thus, indirectly honours Sarah and Elizabeth Fitton, for whom *Fittonia* was named. In some cases, a replacement name (i.e. a name coined to replace a generic name that is outside the rules of the ICN) is coined honouring the same person as the original generic name; in these cases, two generic names, both honouring the same woman, apply to the same genus. These names, too, were treated as “indirect” eponymy with a reference in the column *eponymy-derived-indirectly*, since both generic names refer to the same taxonomic entity.

Wikidata items for the plant genera were updated where necessary or, in a few cases, newly created with information about the genus name, the author(s) of the genus, the year of the original publication and these statements were referenced using the original publication. If the protologue were available on BHL, the BHL bibliographic or page number was added to Wikidata, thus creating a digital link improving access to the protologue.

**People**

After the list of genera was established, we ensured all women being honoured had items in Wikidata (Fig. 1, Workflow C). We researched each person and her contributions, plus information on mythological figures where necessary. We enriched existing or created new Wikidata items using this research. Wikidata notability criteria were followed when creating new items. Our work also included disambiguation from other people with identical or similar names (Groom et al. 2020, Groom et al. 2022). We added any relevant identifiers for the woman to her Wikidata item, thus creating connections to other databases. Examples of such identifiers include library identifiers sourced from the Virtual International Authority File (VIAF), the Harvard Index of Botanists identifier, ORCID identifier (ORCID iD) and genealogical database identifiers, such as FamilySearch, WikiTree, Find A Grave and Geni.

An example of a woman whose contribution to science has been amplified with multiple links through this project is Clara Wehl (1833–1901), a German-born Australian botanical collector and sister of famous botanist Ferdinand von Mueller, who honoured her with the genus *Wehlia* F.Muell. About a year after her Wikidata item was created, links and connections added to Wehl's entry ensured she also became a ‘notable’ figure for the purposes of Wikipedia. An article about her was created, making her contributions more visible to users of that platform as well as to the global general public. If the women honoured in our dataset had written scientific publications, we linked the publication Wikidata items to the women’s Wikidata items via the *author* property.

We acknowledge that gender is a social construct; for this research, our statements on who was a woman or female being were based on how a living person publicly expressed their gender (e.g. through pronoun use). For deceased women or female beings, their implied gender was inferred (perceived gender) either through recorded pronoun use or gender statements in secondary sources. There were two levels of confidence in assigning gender to the person being honoured. High confidence arose when we could cite a source (the protologue or another published source), had direct confirmation from the author of the genus (such as with *Lihengia* Y.S.Chen & R.Ke) or knew the person honoured ourselves. If missing, the perceived gender was added to Wikidata.

We also included, but with less confidence, genera that appeared to have been named for women. Here, we recognised a genus name as likely honouring a woman, but had no direct evidence, such as a female pronoun or feminine grammar in some languages, given in the protologue. For example, the protologue for *Joycea* H.P.Linder merely states “a new genus, named *Joycea* after Joyce Vickery, who did such excellent work on the Australian grasses” (Linder and Verboom 1996: 606). In these cases, further biographical research was done to confirm that the eponymy honoured a woman. In addition, primary references for some names such as *Genlisia* Rchb. and *Pandorea* Spach, presumed to be named for the 18^th^ century Madame de Genlis and the mythical woman Pandora, respectively, were lacking. We, therefore, cited Burkhardt (2022) in Wikidata for these names. Burkhardt is continuing to update her list (pers. comm.) and some sources may be available after publication of this paper.

Occasionally, we found errors of attribution that had originated from our source data that we had entered into Wikidata as *named after* statements. In these cases, we left the original statement in the Wikidata item, but corrected the error by deprecating the original statement with a reference to the source of the correction. We then added a correct/new source of the etymology as a supporting reference for an additional *named after* statement. This happened for the genus *Aster* L., at first attributed to the goddess Astraea (Burkhardt 2018), but corrected to being named for its ‘star shape’ as there is no concrete evidence of its being named for Astraea rather than for its resemblance to a star.

We researched occupations of the honoured women and added or updated statements in Wikidata using the property *occupation*. Many of the more historic women did not have a formal education or a paid profession, but were still active and productive in various fields. This information can be used to analyse, for example, the group of female honourees and prevalent professions or occupations.

Our work also included enriching Wikidata items of taxon authors and compiling certain data to answer specific research questions. IPNI and Tropicos were searched for these author names first and then websites such as BHL, the Global Biodiversity Information Facility (GBIF) or other specialist databases were consulted. We compiled information, such as the nationality and gender of the taxon authors, as well as their relationships to the women after whom they named the genera.

Perceiving the gender of plant name authors was more challenging in the case of names in certain languages and cultures. In these cases, we searched for additional information about the person or contacted colleagues from the respective country. If the genus were recently published, we contacted an author directly if possible. Newly-obtained data were added to Wikidata items of the taxon authors using referenced statements and updated in the “Gendered Author List International Plant Names Index 2020” (Lindon et al. (2020) and subsequent updates to the dataset).

## Geographic coverage

### Description

**Results**

Another publication analysing and discussing the dataset in detail is forthcoming. What follows is a brief summary of results. Our initial query in Wikidata resulted in about 40 plant genera named for women. As a result of our project, we have now a dataset of 728 genera honouring women or female beings. This was a nearly twenty-fold increase in the number of genera linked to women. A total of 31 genera were named after their similarity to other genera that were named for women, i.e. their eponymy is “indirect”.

**Geographic coverage**

**Figure 2.**
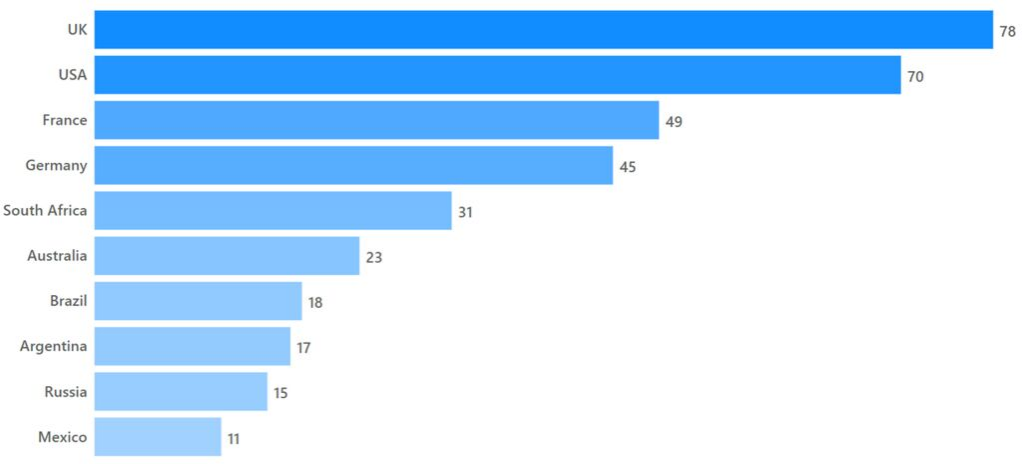
Geography of women honoured. Top 10 countries by count of genera named after non-mythical women associated with that country. Total genera: 441 (where geographical affiliation was known). Total countries represented: 56.

Since we looked at all angiosperm genera named for women or female beings, our geographic coverage was automatically global and the plants included are distributed in all parts of the world. Nevertheless, there is bias related to the countries in which the honoured women were born and/or worked. There are 56 distinct countries associated with the women (country of birth and/or other close association) in our dataset: the ten most commonly occurring (see Fig. 2) are present in roughly 73% of all naming events (see definition of field *eponymy-country* in the Data dictionary, see Column field and description below).

## Taxonomic coverage

### Description

**Figure 3.**
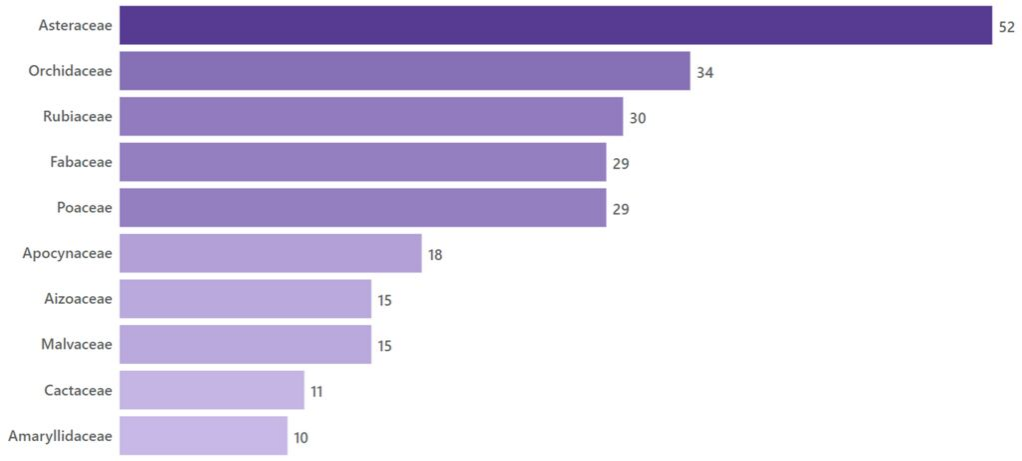
Taxonomic coverage by family. Top 10 families of flowering plants with the count of genera honouring non-mythical women. Total genera: 728. Total families: 134.

The dataset includes 134 distinct angiosperms families that have at least one genus whose name honours a woman. As expected, the largest number of eponyms honouring women, both mythical and non-mythical, corresponds to the most taxon-rich angiosperm families (Fig. 3).

## Temporal coverage

### Notes

**Figure 4.**
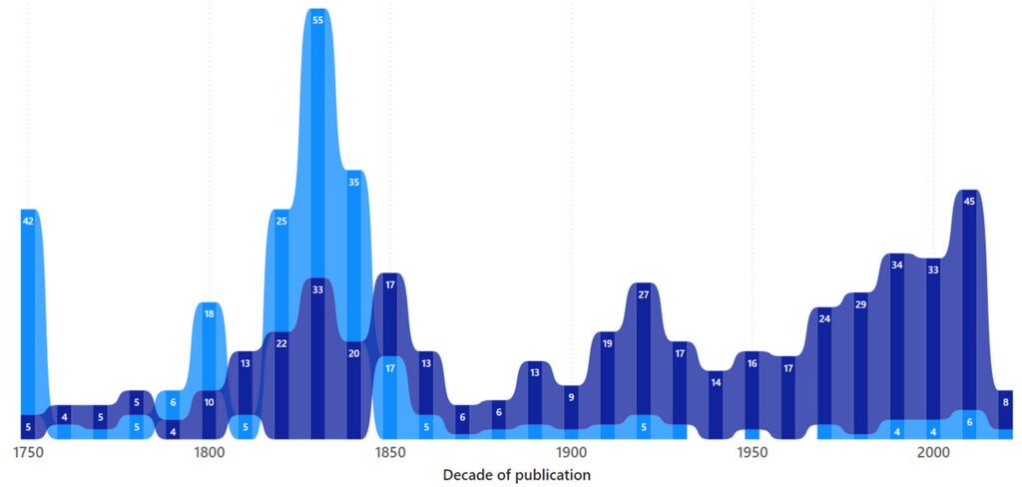
Graph showing count of names by decade of publication. The light blue shows the number named for mythical women and dark blue for non-mythical women honoured.

The dataset covers genera published between 1753 (the starting point for botanical naming, Turland et al. (2018)) to 2023 - a period of 270 years. Fig. 4 shows the number of genera named after mythical versus non-mythical women over time.

## Usage licence

### Usage licence

Creative Commons Public Domain Waiver (CC-Zero)

### IP rights notes

For published dataset: Creative Commons Public Domain Waiver (CC-Zero)

For published paper: Creative Commons Attribution 4.0 International (CC BY 4.0)

## Data resources

### Data package title

Angiosperm genera named for women

### Number of data sets

1

### Data set 1.

#### Data set name

WomenPlantGenera (WPG)

#### Data format

csv

#### Character set

UTF-8

#### Download URL

https://zenodo.org/doi/10.5281/zenodo.8397084

#### Data format version

1.1

#### Description

The dataset provides data for 728 flowering plant genera that were named for women and female beings. Data on the plant genera includes information about the taxon authors, the place and year of publication, identifiers in relevant databases (IPNI, Wikidata, World Flora Online). The dataset also provides further information about the women honoured, including Wikidata identifier, year of birth and death as well as lifespan, occupation, relationship to the authors, if known and links to Wikipedia pages and Bionomia profiles, if applicable. In addition, links to the original publications are provided, if these are available online and the dedication text from the original publication of the genus is cited. Most of this information was also made available in Wikidata, thus providing a Linked Open Dataset.

**Data set 1.** 

Column label	Column description
id	Internal dataset identifier.
genus-name	The full scientific name of the flowering plant genus (herafter 'genus').
genus-qid	Wikidata identifier for the genus name.
genus-qid-url	Wikidata concept URI link for the Wikidata item for the genus name.
genus-ipni-id	International Plant Names Index (IPNI) identifier for the genus name.
genus-ipni-id-url	IPNI link for the genus name.
genus-wfo-id	World Flora Online (WFO) identifier for the genus name.
genus-wfo-id-url	WFO link for the genus name.
genus-wfo-status	The status of the use of genus-name as a label for a taxon, according to the World Flora Online (WFO).
genus-powo-status	The status of the use of genus-name as a label for a taxon, according to Plants of the World Online (POWO). Where the genus status is not recorded in POWO, the field is set to null.
author-std-abbreviation	The authorship information for genus-name formatted according to botanical convention.
author1-qid	Wikidata identifiers for the author(s) of the genus name. [author1]
author2-qid	Wikidata identifiers for the author(s) of the genus name. [author2 if applicable]
author3-qid	Wikidata identifiers for the author(s) of the genus name. [author3 if applicable]
author4-qid	Wikidata identifiers for the author(s) of the genus name. [author4 if applicable]
author5-qid	Wikidata identifiers for the author(s) of the genus name. [author5 if applicable]
author1-qid-url	Concept URI link to Wikidata entry for author(s) of the genus name. [author1]
author2-qid-url	Concept URI link to Wikidata entry for author(s) of the genus name. [author2 if applicable]
author3-qid-url	Concept URI link to Wikidata entry for author(s) of the genus name. [author3 if applicable]
author4-qid-url	Concept URI link to Wikidata entry for author(s) of the genus name. [author4 if applicable]
author5-qid-url	Concept URI link to Wikidata entry for author(s) of the genus name. [author5 if applicable]
author-country	The country or countries closely affiliated with the genus author(s), except for "ex" authors who are "honorary". May include both country of citizenship and if applicable another relevant country, for example, of residence.
author-gender	Indication of perceived of gender of author(s) named in author-name. Length of the string corresponds to the number of authors; sequence of encoded characters corresponds to order of authorship. Each character represents author gender: F = 'female', 'M' = 'male', U = 'unknown'.
author-female-flag	Flag to indicate if at least one of the authors of the genus name is/was a woman.
author-count	Count of people listed in author-std-abbreviation, except for "ex" authors who are "honorary".
protologue-reference	Bibliographic reference to the publication in which the genus name was originally established.
protologue-pub-year	The four-digit year in which the protologue was published. In one case where IPNI provided a range of years, we used the year of publication chosen by POWO.
family-name-wfo	The name of the family under which the genus appears, according to World Flora Online (WFO).
eponymy-derived-indirectly	Flag to indicate that the genus was named after its similarity to another genus, which is itself named after a woman.
eponymy-name	The person or people for whom the genus was named.
eponymy-count	Number of people or mythical figures for whom the genus was named. Where the genus was named after an uncountable group of women, 'n' is used.
eponymy-yob	The birthyear of the person for whom the genus was named. Where known, this is in the form YYYY. Where the year of birth could not be established, the field is set to 'unknown'. Where the person was a mythical figure, the field is set to null.
eponymy-yod	The deathyear of the person for whom the genus was named. Where known, this is in the form YYYY. Where the year of death could not be established, the field is set to 'unknown'. Where the person is living, the field is set to 'current'. Where the person was a mythical figure, the field is set to null.
eponymy-lifespan	Lifespan of the person for whom the genus was named. May include textual indicators of ranges/uncertainty, for example, '1^st^ century BCE'.
eponymy-occupation	Description of the occupation(s) of the person for whom the genus was named. Where the person is a mythical figure, this field holds information on their role/significance.
eponymy-author-relationship	Brief description of the relationship between one or more of the genus authors and the person for whom the genus was named, if applicable.
eponymy-is-real	Flag to indicate that the person for whom the genus named is/has been a living person.
eponymy-is-mythical	Flag to indicate that the person for whom the genus named is a mythical figure.
eponymy-country	The country or countries closely affiliated with the person for whom the genus was named. May include both country of citizenship and, if applicable, another relevant country, for example, of residence. If unknown to authors, the field is set to null.
eponymy1-qid	Wikidata identifier for the woman for whom the genus was named.
eponymy2-qid	Wikidata identifier for a second woman for whom the genus was named, if applicable.
eponymy1-qid-url	Wikidata concept URI link for the Wikidata item for the woman for whom the genus is named.
eponymy2-qid-url	Wikidata concept URI link for the Wikidata item for a second woman for whom the genus is named, if applicable.
protologue-url	URL of a digitised version of the protologue/original publication of the genus. If the origin of the name is not given in the protologue, we link to where the origin of the name was explained. If authors were unable to retrieve a digital version, the field is set to null.
protologue-eponymy-stated	Flag to indicate that there is direct information on eponymy given in the protologue/original publication.
protologue-dedication-text	Verbatim dedication text as recorded in the protologue. In several cases, English translation is provided when clarification was necessary.
eponymy-wikipedia-url	URL of the English Wikipedia page of the person for whom the genus was named. If no URL, a statement was made indicating no English Wikipedia page existed at the time of submission.
eponymy-bionomia-url	URL of the Bionomia page of the person for whom the genus was named. 'n/a' was used for the mythical figures after whom genera were named. Where the person was unlikely to collect natural history specimens, the field was set to null.

## Additional information

**Conclusions**

In addition to the outcome of a comprehensive and novel linked dataset, this work allowed the authors to form new professional and personal links. It enabled networking in botany and data science during the COVID-19 pandemic when travel was not possible and was continued even when travel opened up. We were able to share the work and expense of travelling to conferences to present our results around the world. Specific outcomes included letters of support for funding, research visits to other countries and institutions and professional opportunities or introductions that would not otherwise have happened. All these benefits came about as a result of this long-distance, virtual collaboration. There was also an element of personal satisfaction gained by highlighting these women in a project that, for some of the authors, was conducted outside of our professional duties and responsibilities.

Our research suggests that many of the contributions of the women honoured were in an unpaid role, for example, as a collector of specimens or as a supportive spouse of the author and there was something poetic about choosing to conduct research outside the scope of our day-to-day working life to reveal their stories.

By updating, enriching and openly sharing our data and by communicating openly with the wider community about our research aims and intentions during the research process, we enabled the reuse of those data during our research process. Taking this Open Science approach has ensured we have amplified the contribution of women to the field of botany. We hope to inspire others to use our methods and workflows to empower their own research and follow-up studies on under-represented and under-acknowledged groups in science.

## Supplementary Material

42634B38-A8E9-504A-B378-0B6924B84E35 10.3897/BDJ.11.e114408.suppl1 Supplementary material 1 Examplary SPARQL query for Wikidata Data type SPARQL query (.txt file) Brief description This examplary SPARQL query searches Wikidata for taxa that were named after female botanists, botanical collectors and botanical illustrators. It can easily be adjusted to find all/other taxa named after a specific group of people. Short link to this query: https://w.wiki/4qbL. File: oo_914972.txt https://binary.pensoft.net/file/914972 Leachman S, von Mering S, Shorthouse DP
